# Method of Calculating Desynchronization of DVB-T Transmitters Working in SFN for PCL Applications

**DOI:** 10.3390/s20205776

**Published:** 2020-10-12

**Authors:** Karol Klincewicz, Piotr Samczyński

**Affiliations:** Institute of Electronic Systems, Warsaw University of Technology, Warsaw 00-665, Poland; psamczyn@elka.pw.edu.pl

**Keywords:** passive radar, TDoA, SFN, DVB-T

## Abstract

This paper presents a novel method of calculating desynchronization between transmitters working in a single frequency digital video broadcasting-terrestrial (DVB-T) network. The described method can be a useful tool for enhancing passive radar operations and improving passive coherent location (PCL) sensors to correct their measurements of target localization. The paper presents the problem of localizing DVB-T transmitters utilized by passive radars, and proposes a novel method based on Time Difference of Arrival (TDoA) techniques to solve the problem. The proposed technique has been validated using real signals collected by a PCL sensor receiver. The details of the experiment and extensive result analysis are also contained in this article.

## 1. Introduction

The idea of using non-cooperative transmitters as an illuminator of opportunity (IO) for a passive radar receiver is not a new one. The history of passive radar goes back to the 1930s, when Robert Watson-Watt made the first demonstration of this technology utilizing a BBC radio transmitter as an illuminator of opportunity [1]. The technology was further developed during World War II, when the Germans built a passive radar named Klein Heidelberg which used Chain Home British radars as illuminators of opportunity [1]. After the Second World War, passive radar had no major research interest until the 1990s due to the limited computing power available.

Nowadays, after nearly three decades, passive radar technology is in a stage of maturity. It provides many applications, from air surveillance [2,3,4] to target imaging [5,6], where different kinds of IOs are used, from commercial transmitters (e.g., FM radio, DAB, DVB-T, GSM, among others) [7] to utilize other radars as IOs [8]. Recently, DVB-T transmitters of opportunity became one of the most frequently used as IOs for passive coherent localization (PCL) systems [7,9,10,11], as they are characterized by relatively wide bandwidth (8 MHz) and relatively high power transmitters, which allows for medium range operation up to 100km with a bistatic range resolution of ca. 36 m [7]. DVB-T can operate in multi-frequency network (MFN) or single frequency network (SFN) configurations. One of the problems of an SFN in passive radars is ghost targets. This phenomenon is a result of the number of signal copies acquired by a single receiver.

There are several scientific works in the literature that focus on problems of passive localization systems utilizing SFNs [9,12,13]. A common assumption in most of these works is that SFN transmitters are time-synchronized. In reality, this condition is almost never fulfilled. Most commercial SFN transmitters experience a time shift between each other by design [14]. This fact seems unexplored by passive radar studies, therefore no attempts to cope with it were made. This is unfortunate because SFN desynchronization may introduce major errors in passive radar target localization. The authors of this article recognize a gap in passive radar signal processing studies, and propose a novel method of calculating the time shift between transmitters to compensate for this desynchronization, which might be used in passive radar signal processing to correct target localization error.

In previous publications [15], the authors described a low-cost system designed and developed to localize any source of a signal (an emitter) which might be used for the localization of transmitters of opportunity within the area of passive radar operations. Based on the results of this work, further analysis was made. While positioning a chosen DVB-T transmitter, additional detections occurred through using Time Difference of Arrival (TDoA) techniques. It was discovered that there were two transmitters broadcasting in the SFN, and additional detections were caused both by another transmitter working in the SFN, and the correlation byproduct of two transmitters.

In this article, an analysis of the origin of all the detections is made. Furthermore, the impact of transmitter desynchronization on these detections is explored. Finally, a novel method of calculating the desynchronization between transmitters working in an SFN based on detected TDoA peak positions is presented. The authors believe that this method is a solid addition to any passive radar operating in an SFN, and will be a triggering factor for further studies of the impact of SFN transmitter desynchronization on PCL applications. In the second chapter of this article, passive radar geometry is described. The third chapter provides a brief description of an SFN. In the next chapter, the problem of localizing transmitters used in passive radars is formulated. The next two chapters provide a concise explanation of TDoA. In the seventh chapter, the experiment scenario and setup are presented. In the eighth chapter, the results of the experiment are shown. The next two chapters present the proposed method and impact of measured desynchronization on the passive radar localization of the target. The last chapter concludes this article.

## 2. Passive Radar Geometry

Passive radar operates on a different basis from active radar. In traditional active radar, the signal used for illumination is generated by the transmitting part of the radar system. Most commonly, a single antenna is used for both transmission and reception, switching between those two states. This configuration is described as monostatic. In a passive solution, the transmitter, or in this case the IO, works independently of the radar system. This creates a receiver–transmitter (Rx–Tx) pair working in a bistatic configuration.

**Figure 1:**
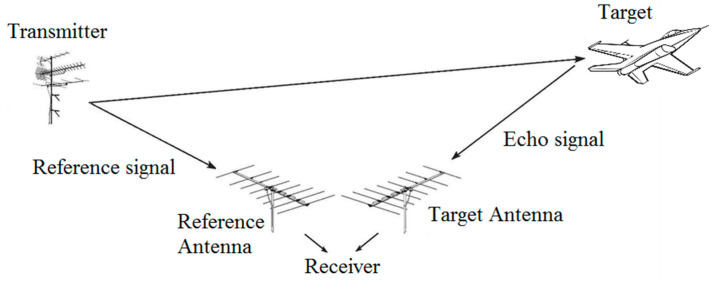
Passive radar principle visualization.

**Figure 2:**
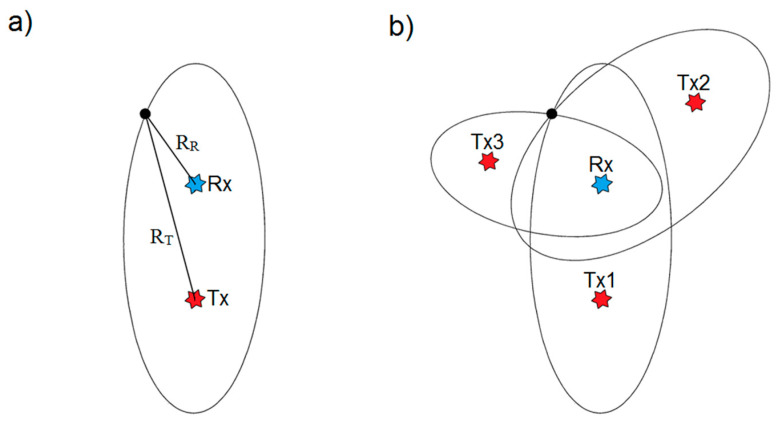
Passive radar geometry: (**a**) for a single ellipse (bistatic configuration), Rx (receiver) and Tx (transmitter) are ellipse foci, and the sum of R_R_ and R_T_ distances is constant; (**b**) by adding more ellipses (multistatic configuration), one can find target localization.

The independence of the exploited transmitter forces each receiver to gather two signals simultaneously: the reference signal gathered from the directional antenna facing the IO; and the measurement signal composed of all echo signals (see Figure 1). Both signals are then cross-correlated, providing the bistatic range and velocity of targets. In terms of localization (with 2D geometry assumption), instead of forming a circle (as in a monostatic configuration), the bistatic pair (Rx–Tx) forms an ellipse on which the target may be found (see Figure 2a). By adding more receivers and/or transmitters, one can form more ellipses. The target is located at the point of their intersection (Figure 2b).

Target localization precision is highly dependent on the accuracy of the transmitter and receiver position data.

## 3. Single Frequency Network Description

An SFN is a network in which all transmitters broadcast the same signal simultaneously. In this article, the DVB-T SFN is at the center of interest. When designing a DVB-T SFN, there are many variables to consider. One of which is the ineliminable problem of interference caused by multiple transmitters broadcasting at the same time in the same frequency. It can be experienced by a DVB-T network user at nearly every position. To prevent any signal loss or intersymbol interference, DVB-T uses Orthogonal Frequency-Division Multiplexing (OFDM) with Guard Interval (GI), preventing data loss in transmission. The values of GI vary between 7μs and 224μs for an 8 MHz channel. This method solves the problem of the multipath for the user, therefore no strict synchronization of transmitters in an SFN, nor precise knowledge of its value is needed [16]. However, for passive radars using DVB-T transmitters as illuminators of opportunity, the exact value of the time shift between transmitters’ broadcasts is valuable information. A slight time difference, assuming the PCL radar system processing the broadcast is using a single transmitter as a reference, will change target localization ellipses. In some cases, this shift is significant enough for PCL processing algorithms not to consider the given ellipses as intersecting within an acceptable error margin, and the measurement will be discarded as non-conclusive.

## 4. Transmitter Localization in Passive Radars

As mentioned in previous sections, the precise localization of illuminators of opportunity in passive radars is of utmost importance. Any inaccuracy is propagated onto target localization. If the positions of the ellipses’ focal points (Rx and Tx) are uncertain, the whole ellipse position may vary. In extreme cases, when two or more ellipses cross on sections nearly parallel to each other, a slight ellipse shift may result in a disproportionately large target localization error, or the rejection of the measurement.

There are many databases provided by broadcast infrastructure operators, government agencies, and noncommercial websites, although transmitters’ positions are frequently inaccurate, out of date, or simply missing. In order to validate data acquired from external sources, it is possible to use a TDoA-based algorithm [15]. Since passive radars commonly consist of more than one receiver [17], in most cases, no additional hardware is needed to gather data for TDoA processing.

## 5. TDoA Geometry

TDoA is a technique used in multilateration localization. Multiple receiver stations gather signals from a single emitter. Considering two-dimensional space, the distance between the nth station position Rx_n_ (x_Rxn_, y_Rxn_) and the emitter position (x_Tx_, y_Tx_) can be described as

(1) $$D_{n}=\sqrt{{\left(x_{Rxn}-x_{Tx}\right)}^{2}+{\left(y_{Rxn}-y_{Tx}\right)}^{2}}$$

Measuring the TDoA between two stations gives information about the difference of distances between the emitter and corresponding stations:

(2) $$t=\frac{D_{n}-D_{m}}{c},$$

where *c* is the speed of light.

**Figure 3:**
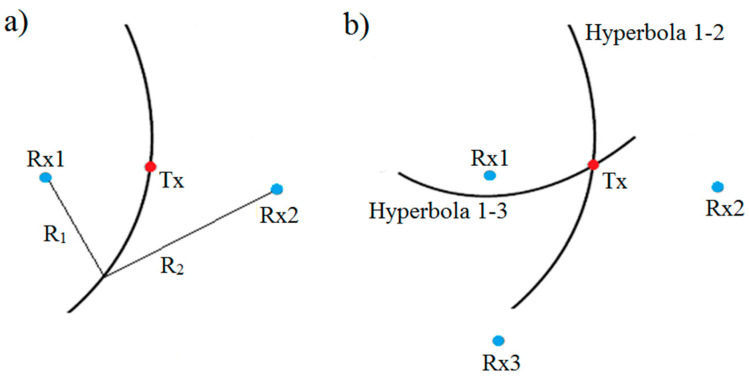
TDoA (Time Difference of Arrival) geometry: (**a**) for single hyperbola pair of receivers (Rx1 and Rx2), Tx are hyperbola foci and the difference of R_1_ and R_2_ distances is constant; (**b**) by adding more hyperbolae one can find the localization of the source of emission.

The TDoA from single-pair receivers forms a hyperbola on which the source of emission is located (see Figure 3a). By adding more receivers, a set of non-linear equations is created. The solution of this set is all the possible locations of the emitter. The problem of solving this equation is well known, and multiple approaches have been proposed [18].

For any number of receivers, N, greater than one, it is possible to create a set of N-1 equations and form N-1 hyperbolae (Figure 3b).

The exact value of the TDoA between two stations (Rx_n_ and Rx_m_) is calculated by cross-correlating the acquired continuous signals (s_n_ and s_m_, respectively). Cross-correlation is a mathematical tool used in signal processing to analyze similarities between two signals in the function of time delay (τ). For continuous signals, cross-correlation can be written as

(3) $$C_{nm}\left(\tau \right)=\overset{\infty }{\underset{-\infty }{\int }}s_{n}^{*}\left(t\right)\;s_{m}\left(t+\tau \right)\;\mathrm{dt},$$

where * denotes complex conjugation. The expected result of cross-correlation in a bistatic configuration should consist of a distinct peak corresponding to the TDoA value, t.

## 6. TDoA in SFN

**Figure 4:**
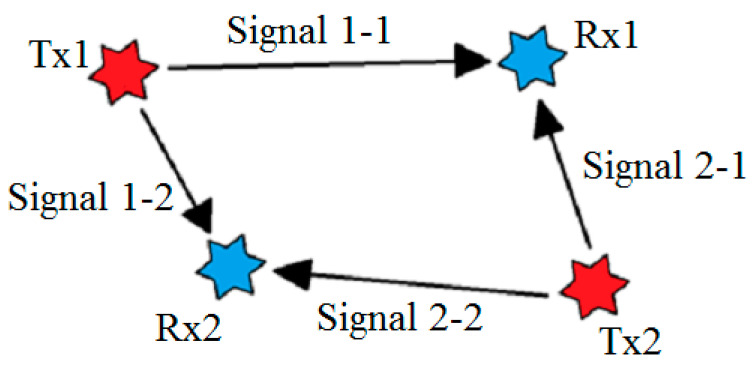
SFN (single frequency network) signal propagation. Assuming the geometry of two receivers and two transmitters, there are four different signals to consider in cross-correlation analysis.

In the case of multiple emitters working in an SFN, the results of cross-correlation are different. Considering two SFN fully-synchronized transmitters as sources of emission, more than one peak will occur. Since each receiver acquires the same signal from two transmitters simultaneously (see Figure 4), there are four possible peaks on cross-correlation:

1. Correlation between Signal 1-1 and Signal 1-2. This peak is the result of the TDoA for the signal emitted by Tx1.
2. Correlation between Signal 2-1 and Signal 2-2. This peak is the result of the TDoA for the signal emitted by Tx2.
3. Correlation between Signal 1-1 and Signal 2-2. This peak is a ghost detection. No physical interpretation.
4. Correlation between Signal 1-2 and Signal 2-1. This peak is a ghost detection. No physical interpretation.

Assuming that the precise position of transmitters (x_Ti_, y_Ti_) is well known, the calculation of the exact time of occurrence for all four peaks is possible. Writing distance between point A(x_A_, y_A_) and point B(x_B_, y_B_) as

(4) $$R_{A-B}=\sqrt{{\left(x_{A}-x_{B}\right)}^{2}+{\left(y_{A}-y_{B}\right)}^{2}},$$

and the times of appearance for all described peaks are

(5) $$t_{1}=\frac{R_{Rx1-Tx1}-R_{Rx2-Tx1}}{c}\;t_{2}=\frac{R_{Rx1-Tx2}-R_{Rx2-Tx2}}{c}\;t_{3}=\frac{R_{Rx1-Tx1}-R_{Rx2-Tx2}}{c}\;t_{4}=\frac{R_{Rx2-Tx1}-R_{Rx1-Tx2}}{c}$$

Through the comparison of the calculated values and the real results of cross-correlation, it is possible to compute:

- The TDoA measurement error in the case of t_1_ and t_2_
- The sum of the TDoA measurement error and the transmitters’ synchronization accuracy in the case of t_3_ and t_4_.

Synchronization accuracy information can be extracted due to the fact that the peaks at t_3_ and t_4_ are results of the correlation of two signals each from two different transmitters. Based on this fact, the authors have proposed an algorithm to determine the desynchronization time between DVB-T transmitters, which is described in the next section of this paper.

## 7. TDoA in SFN

**Figure 5:**
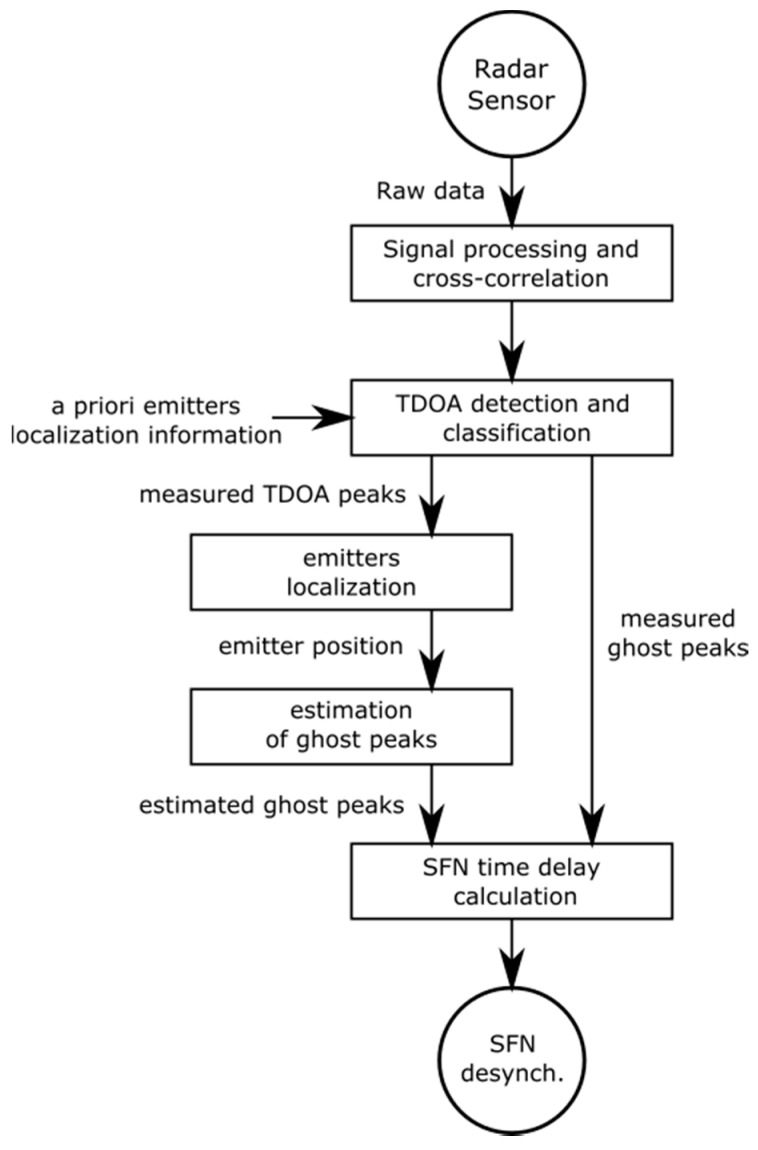
Proposed method of calculating the desynchronization of transmitters working in an SFN.

The proposed algorithm for calculating the desynchronization of DVB-T transmitters working in an SFN is shown in Figure 5.

All the stages of the algorithm can be described in the following way: in the first step, after acquiring raw data from radar sensors and applying initial signal conditioning (e.g., decimation, pre-filtration, windowing), data from each pair of sensors are cross-correlated. Then, based on a priori knowledge of the localization of emitters, the maxima of the cross-correlation functions are identified. The peaks corresponding to the possible locations of emitters are marked as TDoA peaks. The rest of the peaks are classified as ghost peaks. The next step is to calculate the emitters’ precise localization using measured TDoA peaks. After this step, 3D positions of all emitters are determined. Based on the estimated Tx’s positions and known Rx position, the values of ghost peaks are estimated. Finally, through the comparison of the calculated and measured values of the ghost peaks, the estimated value of the desynchronization between emitters can be found. In the next section, the validation of the proposed algorithm using real measurements is presented.

## 8. Experiment Setup

**Figure 6:**
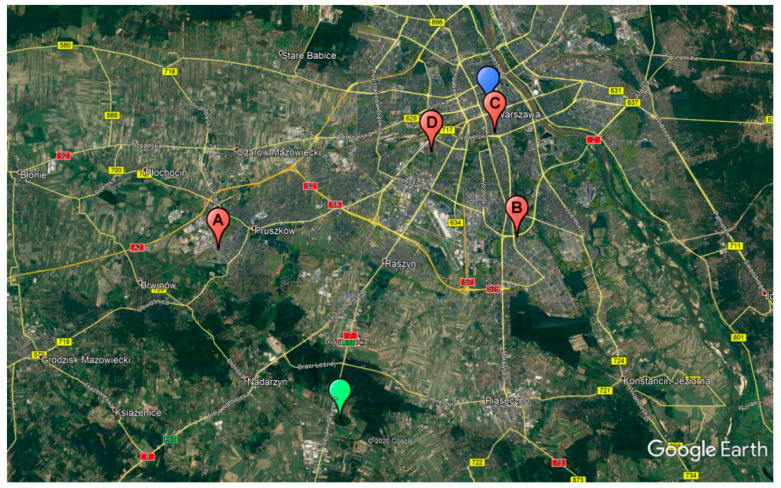
Map showing positions of used transmitters of opportunity (Raszyn—green marker, PKiN—blue marker) and surveillance stations (red markers).

**Figure 7:**
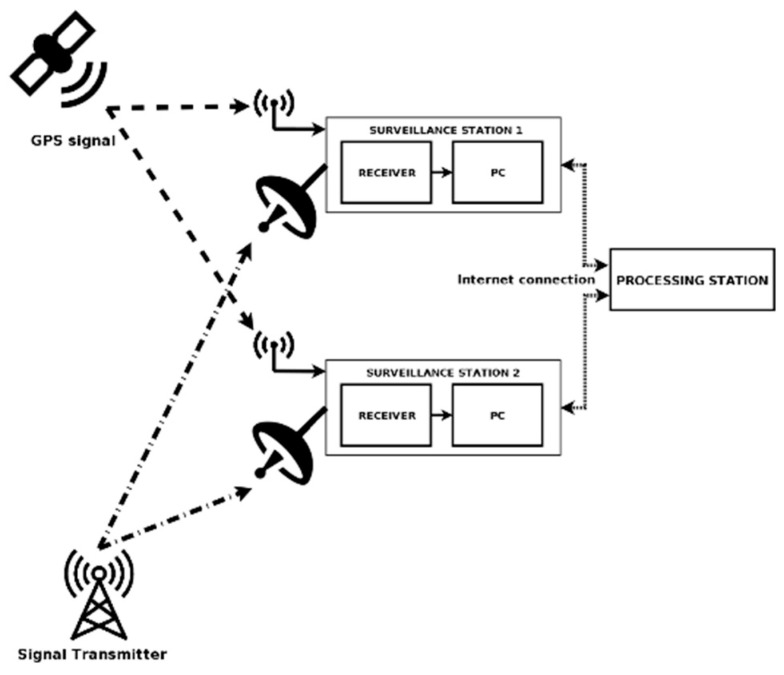
A simplified scheme of two surveillance stations. In the described experiment, four stations were used.

To validate the claim that the synchronization accuracy of two transmitters working in an SFN can be calculated using only passive radar receivers, an experiment was conducted. Four surveillance stations imitating passive radar receivers were placed in different locations in Warsaw and Pruszków, Poland (Figure 6). Each of the stations consisted of a receiver based on a software defined radio (SDR), a wide-angle antenna, and a portable personal computer (PC) used as a communication and storage device (Figure 7). Due to the researchers having previous experience [15] with it, the B210 Universal Software Radio Peripheral (USRP) platform was chosen to fulfill the role of a receiver. It is capable of collecting a signal with 56 MHz of instantaneous bandwidth, covering frequencies from 70 MHz up to 6 GHz.

The receivers used a GPS signal to achieve 50 ns (RMS 1-Sigma) synchronization accuracy and 10-m position accuracy. All were set to collect data on the 690 MHz central frequency and 10 MHz bandwidth, which covers the entire 48th DVB-T channel.

The stations were controlled remotely via an internet connection provided by a PC. All the data collected by the receivers were transferred to a central processing station.

Two DVB-T transmitters operating in an SFN were chosen as illuminators. Both transmitters were located in Warsaw, Poland. The main parameters of both transmitters are shown in Table 1.

It is important to mention here that the precise localization of both radio transmitter towers is ambiguous. Several sources from on-line databases provided different information (see Table 2). Taking the PKiN transmitter as an example, these differences varied over 40 m. As mentioned before, such an error will influence any passive radar localization attempts.

Choosing Wikipedia.org as a source of the transmitters’ positions, and gathered GPS data for the receivers’ positions, all distances were calculated. All previous calculations (5) were expanded by a z-factor to match a three-dimensional scenario. The results of these calculations, recalculated from time units into distance units and rounded to one meter, are presented in Table 3.

## 9. Experiment Results

**Figure 8:**
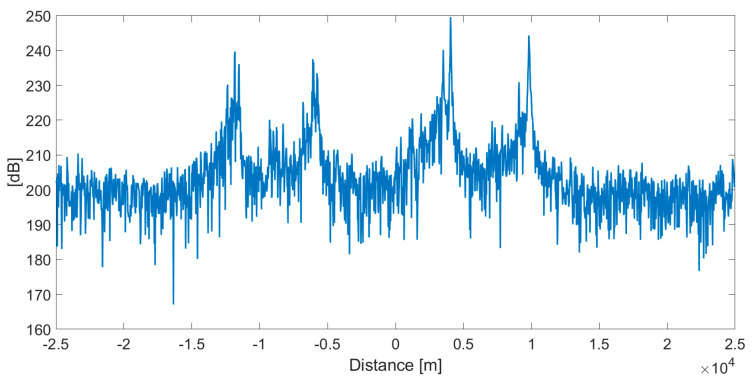
Example of cross-correlation results calculated for stations 1-2 pair. Four distinct areas of interest with correlation peaks can be seen.

A series of measurements were conducted with the described system. After initial signal processing, cross-correlation was calculated for each pair of receivers (example for stations 1-2 pair in Figure 8). As predicted, in every cross-correlation result, four distinct maxima were observed.

Next, the data were processed to extract local maxima corresponding to TDoA peaks and ghost detection peaks. For every measured peak, a comparison to the calculated value was made. For TDoA peaks, the mean difference between the calculated and measured was equal to 30m, with the maximum values never exceeding ±150 m (Table 4). This difference can be explained as the sum of two sources of error: receiver synchronization error (15 m RMS 1-sigma), and the positioning error of receivers and transmitters.

Meanwhile, the differences were significantly higher for ghost peaks and oscillated around ±1100 m (Table 5). There is clearly another source of error.

This disparity can be explained by the time delay in transmission between two transmitters. To find the value of this delay, the algorithm described in Chapter 7 was used. Using the measured TDoA peaks, the Cartesian positions of the transmitters were found. Based on the Cartesian positions of the transmitters, the assumed positions of the ghost peaks were calculated. Lastly, by subtracting this value from the measured ghost peak positions, a desynchronization between the emitters was found. In this case, based on multiple short measurements spanning within 1 h, it can be approximated to be 3.63 μs, meaning that the Tx2 broadcast was lagging behind Tx1. This made a lot of sense, since this value minimized the SFN multipath effect for DVB-T recipients in the city of Warsaw. Further tests have shown that, for the two transmitters chosen, this value is relatively stable over a few hours but can vary over a few days, thus a time delay calculation should be made every time in a PCL system before using DVB-T transmitters as illuminators of opportunity.

## 10. Desynchronization Impact on PCL

In this section, the impact of desynchronization on target location in passive radar is studied. For this purpose, a scenario is considered where a passive radar is operating in an SFN environment and only one reference signal is gathered. Such a case is realistic when the quality of a reference signal from a second transmitter is poor, due to: the lack of an unobstructed line of sight between the emitter and receiver; a strong leak of the dominant transmitter into a weaker transmitter reference signal; or potentially other causes. Assuming that the radar signal processing algorithm takes into account the time delay between the two reference signals reaching the receiver, it is perfectly valid to gather only one reference signal when utilizing transmitters in SNF. In such a scenario, it is even possible to localize a target with a single receiver. However, this approach is highly susceptible to any transmitter desynchronization.

To show how desynchronization affects passive radar localization errors in a single reference signal scenario, a set of simulations was performed. In each of the simulations, the positions of both transmitters and all receiver stations remained the same as in the experiment described in Section 8. Transmitter desynchronization was set to a value of 3.63 μs, as estimated in the experiment. Lastly, the position of the target was randomly chosen within 50 km from the midpoint of the transmitters. For each of the four receivers, a pair of ellipses can be found. Each ellipse corresponds to the signal transmitted by one of the emitters and is reflected by the target. With two ellipses, it is possible to find the point of their interception. The final result of the simulation was a distance measured in meters between the intersection point and the actual target position. When the desynchronization of the transmitters was set to zero, this distance was shorter than 10^−4^ m. The non-zero value is a product of numerical errors of the algorithm responsible for finding the intersection point.

**Figure 9:**
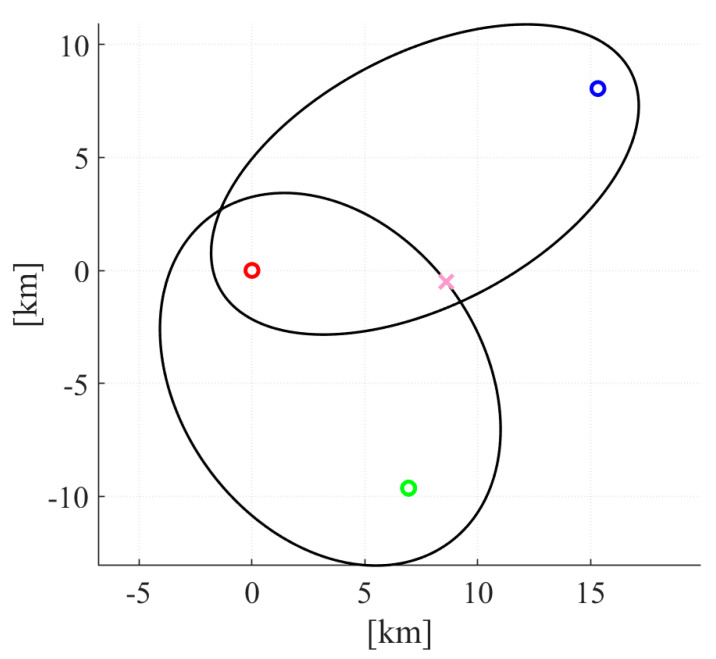
Visual example of a single simulation for station 4 (red marker) and two transmitters (Raszyn—green marker, PKiN—blue marker). Target’s position (marked with pink cross) does not match the ellipses intersection point due to transmitter desynchronization.

A total of 10^4^ simulations for each surveillance station using the Monte Carlo method were made, each with a different target position. A visual example of a simulation is presented in Figure 9, and the simulation results are summarized in Table 6.

Depending on the geometry of the target and receiver, desynchronization of transmitters equal to the measured 3.63 μs can introduce on average a 2 km error in passive radar localization for the geometry assumed in the simulation. In extreme cases, the error can reach over 6 km. Using the proposed method, the desynchronization error can be eliminated or greatly mitigated.

## 11. Conclusions

As shown, the synchronization of SFN DVB-T transmitters (and possibly other commercial transmitters) is not precise. While it is not a problem for a commercial user, it may be a source of significant localization error for a passive radar system.

The described experiment proved not only that the chosen DVB-T transmitters were desynchronized by 3.4 μs, but also that the synchronization error of two transmitters working in an SFN can be calculated using only passive radar receivers, without any additional hardware systems. Such a method is recommended, especially when a priori knowledge of transmitters’ localizations is not reliable or inaccurate. This additional information about desynchronization can be used to alter the localization algorithms of passive radars, and possibly further improve their precision and detection probability, which the authors intend to implement in a future PCL processing chain.

## Figures and Tables

**Table 1:** Transmitter information.

Tx name	Latitude *	Longitude *	Channel	Power
RTCN Raszyn	52.0733 N	20.8855 E	48 (690 MHz)	100 kW
RTCN PKiN	52.2317 N	21.0064 E	48 (690 MHz)	3 kW

* source: Wikipedia.org.

**Table 2:** Transmitter localization differences.

Source	Latitude of PKiN	Longitude of PKiN
FMSCAN.org	52.23166 N	21.00611 E
DVBTmap.eu	52.23172 N	21.00675 E
Google Earth	52.23184 N	21.00599 E
Wikipedia.org	52.23167 N	21.00639 E

**Table 3:** Distances between receivers and transmitters.

Distances [m]	Station 1	Station 2	Station 3	Station 4
Tx1—RTCN Raszyn	18,481	14,423	16,038	11,882
Tx2—RTCN PKiN	1486	7517	4098	17,306

**Table 4:** TDoA Peaks.

TDoA Peaks [m]	Tx1 Calc.	Tx1 Meas.	Tx1 Diff.	Tx2 calc.	Tx2 Meas.	Tx2 Diff.
Stations 1-2	4058	4080	−22	−6031	−6060	29
Stations 1-3	2443	2460	−17	−2612	−2520	−92
Stations 1-4	6599	6510	89	−15,820	−15,870	50
Stations 2-3	−1615	−1590	−25	3419	3390	29
Stations 2-4	2541	2430	111	−9789	−9840	51
Stations 3-4	4156	4020	136	−13,208	−13,230	22

**Table 5:** Ghost Peaks.

Ghost (Gh.) Peaks [m]	Gh.1 Calc.	Gh.1 Meas.	Gh.1 Diff.	Gh.2 Calc.	Gh.2 Meas.	Gh.2 Diff.
Stations 1-2	10,964	9840	1124	−12,937	−11,820	−1117
Stations 1-3	14,383	13,230	1153	−14,552	−13,410	−1142
Stations 1-4	1175	30	1145	−10,396	−9390	−1006
Stations 2-3	10,325	9180	1145	−8521	−7350	−1171
Stations 2-4	−2883	−3810	927	−4365	−3330	−1035
Stations 3-4	−1268	−2460	1192	−7784	−6870	−914

**Table 6:** Simulation Results of Localization Error.

	Station 1	Station 2	Station 3	Station 4
mean error [m]	1.892 × 10^3^	1.954 × 10^3^	1.913 × 10^3^	2.091 × 10^3^
variance [m^2^]	1.495 × 10^6^	1.558 × 10^6^	1.547 × 10^6^	1.431 × 10^6^
